# Biparental and Androgenetic Somatic Mosaicism with Presentation of Non-Syndromic Severe Neonatal Hyperinsulinemia

**DOI:** 10.3390/ijms26167985

**Published:** 2025-08-19

**Authors:** Miguel Angel Alcántara-Ortigoza, Marcela Vela-Amieva, Ariadna González-del Angel, Miriam Erandi Reyna-Fabián, Liliana Fernández-Hernández, Bernardette Estandía-Ortega, Sara Guillén-López, Lizbeth López-Mejía, Isabel Ibarra-González, María de la Luz Ruiz-Reyes, Raúl Calzada-de León, Mauricio Rojas-Maruri, Flora Zárate-Mondragón, Go Hun-Seo, Hane Lee, Cynthia Fernández-Lainez

**Affiliations:** 1 Laboratorio de Biología Molecular, Instituto Nacional de Pediatría, Secretaría de Salud, Mexico City 04530, Mexico; malcantaraortigoza@gmail.com (M.A.A.-O.); ariadnagonzalezdelangel@gmail.com (A.G.-d.A.); erandif@yahoo.com (M.E.R.-F.); dralilianafernandez@gmail.com (L.F.-H.); bernsestandia@yahoo.com.mx (B.E.-O.); 2 Laboratorio de Errores Innatos del Metabolismo y Tamiz, Instituto Nacional de Pediatría, Secretaría de Salud, Mexico City 04530, Mexico; dravelaamieva@yahoo.com (M.V.-A.); guillen.lopez.sara@gmail.com (S.G.-L.); lizbeth712@hotmail.com (L.L.-M.); 3 Unidad de Genética de la Nutrición, Instituto de Investigaciones Biomédicas, Universidad Nacional Autónoma de México, Mexico City 04530, Mexico; icig@servidor.unam.mx; 4 Servicio de Endocrinología, Instituto Nacional de Pediatría, Secretaría de Salud, Mexico City 04530, Mexico; luceroruiz15@yahoo.com (M.d.l.L.R.-R.); raulcalzada@yahoo.com (R.C.-d.L.); 5 Departamento de Patología, Instituto Nacional de Pediatría, Secretaría de Salud, Mexico City 04530, Mexico; romace1950@yahoo.com.mx; 6 Departamento de Gastroenterología y Nutrición, Instituto Nacional de Pediatría, Secretaría de Salud, Mexico City 04530, Mexico; florazarate@gmail.com; 7 3billion, Inc., Seoul 03161, Republic of Korea; ghseo@3billion.io (G.H.-S.); hlee@3billion.io (H.L.)

**Keywords:** androgenetic/biparental mosaicism, DNA profiling, congenital hyperinsulinism, mosaic genome-wide paternal uniparental disomy, nesidioblastosis, neonatal hypoglycemia, regions of homozygosity (ROH), whole-exome sequencing (WES), genome-wide paternal uniparental isodisomy (GWpUPID)

## Abstract

Genome-wide paternal uniparental isodisomy mosaicism (GWpUPIDM) is an extremely rare condition characterized by varying proportions of an androgenetic cell line across different tissues. It is primarily associated with severe congenital hyperinsulinism (CHI), Beckwith–Wiedemann syndrome (BWS) stigmata, a high risk (69–79%) of developing neoplasia and, in some cases, additional manifestations of multilocus paternal imprinting disorders (MPIDs). We herein report the first Mexican/Latin American female patient GWpUPIDM presenting with non-syndromic CHI requiring subtotal pancreatectomy and persistent but unexplained asymptomatic diffuse hepatopathy. When she was 8.5 years old, whole-exome sequencing (WES) in blood revealed an unexpectedly high (~92%) proportion of regions of homozygosity. DNA profiling confirmed a single haploid set of paternal chromosomes in both biparental and androgenetic cell lines, with varying proportions of the androgenetic lineage in leukocytes (84%), resected pancreas (74%), buccal cells (47%), and hair follicles (0.7%). Additional WES trio analysis using gDNA from the patient’s buccal cells and blood samples from both parents revealed an allelic frequency of ~75% for the paternally inherited variant NM_000158.4(*GBE1*):c.555+1G>T [ClinVar:632422; dbSNP:rs759707498]. At age 8.5, the patient exhibited no clinical features of BWS, MPIDs, or neoplasia. However, she presented persistent hepatic abnormalities that warrant further investigation to rule out an unmasked glycogen storage disease type IV (OMIM#232500). Our findings emphasize the critical need for early diagnosis of GWpUPIDM using SNP-based microarray or WES with further confirmation through DNA profiling in patients presenting with CHI, placental mesenchymal dysplasia, BWS stigmata, or other MPID-related conditions, including neoplasia, to facilitate timely cancer surveillance and management.

## 1. Introduction

The uniparental contribution of a diploid genome (diandry or digyny) in the human zygote is typically a lethal condition, except for the ~30 live-born patients reported in the literature up to 2019 [1]. However, in ChromosOmics Database (https://cs-tl.de/DB/CA/UPD/0-Start.html#Frequ, accessed on 17 June 2025), 153 cases are currently recorded, including miscarriages and molar pregnancies, among others, lacking complete clinical information. Most of these cases exhibit genome-wide paternal uniparental isodisomy mosaicism (GWpUPIDM, ORPHA code: 329813; https://www.orpha.net/en/disease/detail/329813, accessed on 3 March 2025), wherein two diploid cell lines coexist: one with an expected biparental genome contribution and another with an androgenetic genome contribution. Mechanistically, the most plausible proposal is that post-zygotic mitotic errors involving two endoreduplication events of the paternal haploid genome compensate for a maternal endoreduplication failure in a monospermic fertilization model of a normal haploid oocyte [2].

GWpUPIDM is mainly identified in 46,XX individuals with a history of placental mesenchymal dysplasia (PMD) [3,4,5], prematurity [4], or clinical manifestations of paternal imprinting disorders related to the 11p15 region, including isolated hemihypertrophy (OMIM %235000) [4], Beckwith–Wiedemann syndrome (BWS, OMIM #130650) [1], and congenital hyperinsulinism (CHI, pancreatic hyperplasia-nesidioblastosis) with severe neonatal hypoglycemia [4,6]. Additionally, affected individuals present an increased lifetime risk (~79%) of developing adrenal, renal, and hepatic embryonal neoplasia [4]. Affected males are reported to a much lesser extent than their female counterparts (https://cs-tl.de/DB/CA/UPD/0-Start.html#Frequ, accessed on 17 June 2025). Mosaicism involving a normal biparental 46,XY and androgenetic 46,XX cell line, likely generated by dispermic fertilization, could be detected prenatally in such patients due to placental abnormalities. If mosaicism is confined to the placenta, then, normally, live-born males can be expected [7,8]. Moreover, clinical manifestations among symptomatic males and females seem to be similar [1,9,10], although the level of risk of developing disorders of sexual development and germ cell tumors in mosaic 46,XX/46,XY males remains to be determined [1].

Depending on the degree of mosaicism, clinical manifestations may extend beyond BWS to other imprinting disorders resulting from paternal uniparental disomy, including those of chromosome 6 (diabetes mellitus, transient neonatal, type 1; OMIM #601410), chromosome 14 (Kagami–Ogata syndrome; OMIM #608149), chromosome 15 (Angelman syndrome or AS; OMIM #105830), and chromosome 20 (pseudohypoparathyroidism type Ib; OMIM #603233) [1,4,11,12]. Furthermore, paternal uniparental isodisomy (pUPID) may unmask autosomal recessive disorders [13,14,15,16]. Summarized major clinical findings in 29 live-born infants with GWpUPIDM published up to 2019 were described by Sheppard et al. [1].

The estimated prevalence of GWpUPIDM is <1/1,000,000 individuals (https://www.orpha.net/en/disease/detail/329813, accessed on 1 April 2025). However, it has been reported in 1.8% (N = 29/1633) of gestational losses due to suspected molar pregnancy [5], 0.02% of patients undergoing whole-exome sequencing (WES) or SNP-based chromosomal microarray analysis [17], and up to 8% of patients with BWS, particularly those with atypical phenotypes involving a history of prematurity, multilocus paternal imprinting disorders (MPIDs), and severe CHI [1].

Here, we describe a Mexican pediatric patient diagnosed with GWpUPIDM, who initially presented CHI and severe neonatal hypoglycemia requiring subtotal pancreatectomy. GWpUPIDM was identified through WES and DNA profiling in various tissues, which revealed a mosaic androgenetic cell lineage. This case highlights the importance of early diagnosis of GWpUPIDM for close follow-up of benign and/or malignant embryonal neoplasms, the potential development of autoimmune conditions [13], and the possibility that autosomal recessive diseases may be unmasked by pUPID.

## 2. Results

### 2.1. Case Presentation

**Figure 1:**
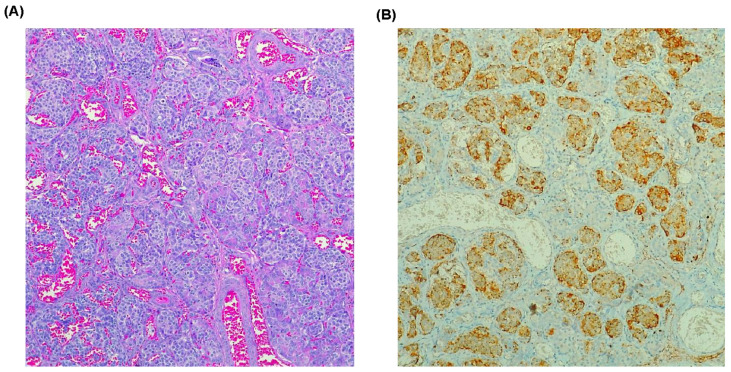
Histopathological findings of resected pancreas (photomicrographs at 10×). (**A**) Hematoxylin and eosin staining reveals an increased number of hyperplastic pancreatic islets. (**B**) Immunohistochemistry with chromogranin, showing that acini are increased in number and size.

The female patient initially presented to our service at 6 years and 3 months old. She was the third child of healthy, non-consanguineous Mexican parents (mother 36 years old; father 37 years old) with no significant medical history related to their daughter’s condition. The pregnancy was complicated by maternal hypoglycemia, pre-eclampsia, and threatened abortion, which was managed with absolute rest and finally resolved via cesarean section at 35 weeks of gestation due to premature rupture of membranes. No information was available regarding placentation. At birth, the patient weighed 2765 g, measured 46 cm in length, and had a head circumference of 34 cm. The APGAR score was 8/9, and Silverman’s score was 4. Soon after birth, she was diagnosed with a discrete frontal hemangioma, and ultrasound evaluation revealed hepatomegaly and diffuse liver disease. Routine metabolic and auditory neonatal screening results were normal. However, the patient developed neonatal sepsis due to *Escherichia coli*; this was successfully treated with meropenem and amikacin and resolved at 16 days of extrauterine life. At 1 month of age, the patient experienced seizures attributed to severe non-syndromic hypoglycemia (blood glucose < 40 mg/dL; reference value 70–100 mg/dL) that was caused by hyperinsulinism (serum insulin 20.8–39.9 uU/mL, ref. 6–27 uU/mL; C-peptide: 2.4 ng/mL; ref. 0.9–4 ng/mL) and proved refractory to pharmacological management. A brain ultrasound showed findings consistent with periventricular hypoxia. To further investigate the etiology of the hyperinsulinism, an abdominal positron emission tomography (PET) scan using Gallium-68 was performed; this study revealed overexpression of the somatostatin receptor in the body and tail of the pancreas, suggesting a diagnosis of nesidioblastosis. Accordingly, within her first 50 days of extrauterine life, the patient underwent a subtotal pancreatectomy (95%) with resection of the head, body, and tail of the pancreas (5 × 1.5 cm^2^, 6 g). This procedure alleviated the hypoglycemic crisis, yielding a serum insulin level of less than 2 uU/mL (ref. 6–27 uU/mL) and a C-peptide level of 0.4 ng/mL (ref. 0.9–4 ng/mL). Histopathological analysis of the resected pancreatic tissue revealed cytologically normal tissue but the presence of an increased number of hyperplastic pancreatic islets characterized by slight enlargement of islets and cell nuclei with prominent nucleoli. Immunohistochemical staining of resected pancreatic tissue was positive for insulin and chromogranin, confirming the diagnosis of diffuse nesidioblastosis (Figure 1).

Post-pancreatectomy, the patient continued to experience hypoglycemia and required intensive glucose management, including bolus administration and continuous intravenous infusion rates of glucose up to 18 g/kg/min. Despite these interventions, the hypoglycemia remained refractory, necessitating a 6-day course of hydrocortisone. Hyperinsulinism was reconfirmed by observation of elevated insulin levels (20.8 and 39.9 µU/mL), and concurrent hypocalcemia required calcium supplementation. Due to the persistent hypoglycemia, diazoxide was initiated at doses ranging from 8 to 15 mg/kg/day. The patient achieved glycemic stabilization at 6 years and 5 months of age and has retained this status to date. Subsequent evaluations, including a thoracoabdominal computed tomography scan, identified hepatomegaly and suprarenal glands with a normal morphology but increased size for her age.

### 2.2. Genetic Evaluation and Clinical Follow-Up

**Figure 2:**
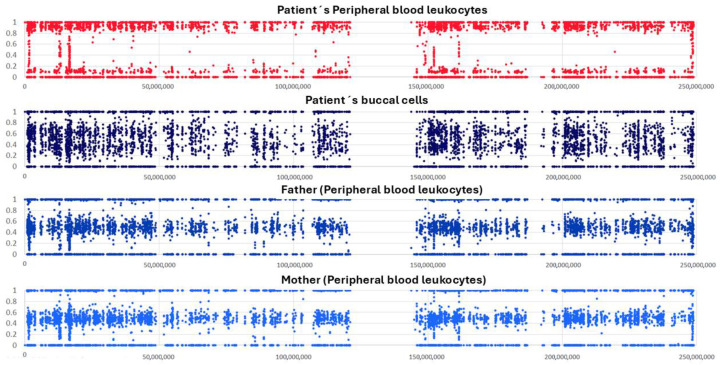
Extreme skewness in allelic frequencies is observed on chromosome 1 for single-nucleotide variants in whole-exome sequencing (WES) data of genomic DNA (gDNA) from the patient’s peripheral blood leukocytes (~92%) but is less pronounced in that from buccal cells (~62%). This phenomenon is observed in all chromosomes. Parental samples, which are presented as controls, show typical normal distribution of variant allelic frequency (0.5) in gDNA samples.

**Figure 3:**
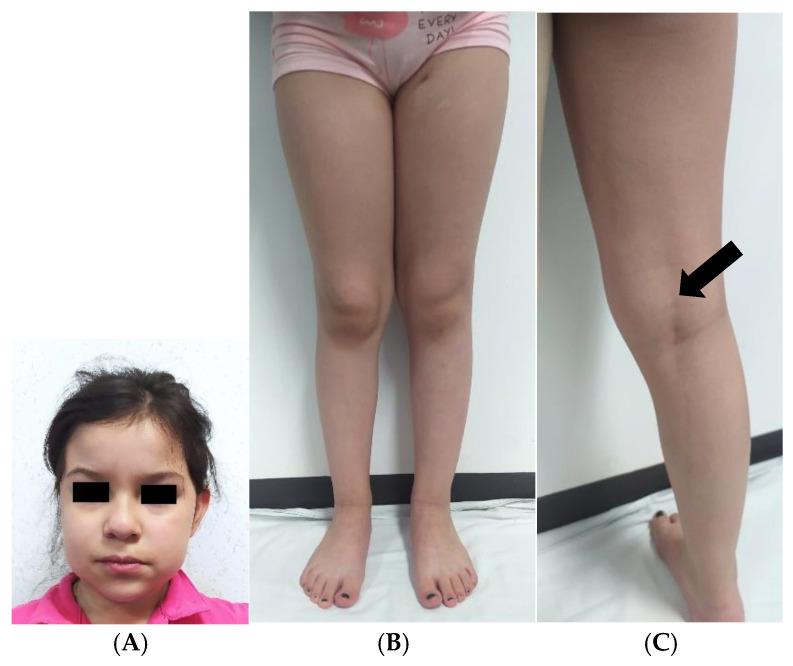
Clinical pictures of the patient at 8.5 years of age. (**A**) Minor facial dysmorphias. (**B**) Very mild hemihypertrophy in the legs. (**C**) Discrete lineal hyperpigmentation on the back of the right leg (arrow). No other BWS stigmata (i.e., facial hemangioma, macroglossia, or abdominal wall defects) were noted.

Due to her history of severe hypoglycemia related to CHI, along with prenatal diffuse hepatomegaly, the patient was invited at 8 years of age to participate in a cohort of Mexican patients with suspected intermediary errors of metabolism, who would undergo WES for genetic diagnosis. This patient’s first WES analysis (previously published as patient ID 3bINP-012) performed with genomic DNA (gDNA) from peripheral leukocytes was considered inconclusive due to the unexpectedly high (~92%) proportion of regions of homozygosity (ROH, Figure 2) [18]. Following this result, additional investigations were conducted to assess the possibility of GWpUPIDM. The first step was a post hoc clinical examination to detect features associated with paternal imprinting disorders, including BWS stigmata and/or embryonal neoplasms. At 8 years and 6 months of age, the patient weighed 19 kg (<1P, Z = −3.23) and had a head circumference of 51 cm (6P, Z = −1.56) and a height of 117 cm (<1P, Z = −2.75). Minor dysmorphisms were observed, including dolichocephaly, a forehead with bifrontal narrowing, full cheeks, and brachydactyly. She also had mild hemihypertrophy in the legs (right and left leg length, 64 cm and 65 cm, respectively; right and left thigh circumference, 38 cm and 37 cm, respectively) but not in the arms. On the back of her right leg, there was a region with mild, lineal hyperpigmentation of the skin. However, no clinical sign of additional MPIDs was identified, such as the earlobe creases, facial hemangioma, macroglossia, or umbilical hernia suggestive of BWS or the wide-spaced teeth, macrostomia, or mandibular prognathy suggestive of AS (Figure 3). An abdominal ultrasound performed at age 8.5 revealed a heterogeneous, granular hepatic parenchyma, suggesting the presence of isolated fibrotic areas and echogenic bile duct walls; these findings were consistent with cholangitis. No other abnormality was detected in the hepatic vasculature, common bile duct, spleen, or kidneys. Hepatic enzymes, bilirubin, and serum alpha-fetoprotein (AFP) levels were within the normal ranges. Although the patient experienced a delay in acquiring motor milestones before the age of 1 year, her neurodevelopment at age 8.5 was appropriate for her age, and she attended school without difficulty.

### 2.3. Genomic DNA Profiling and WES Analysis

**Figure 4:**
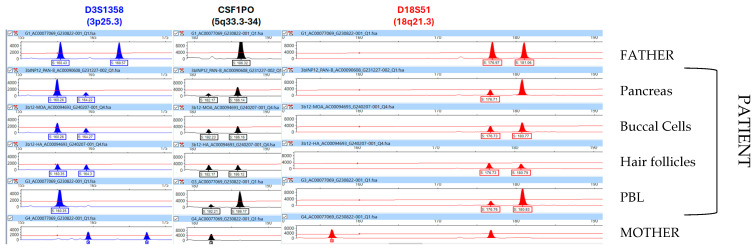
Representative electropherograms of three semi-informative and informative STR markers used to roughly estimate the proportion of the androgenetic cell line in gDNA obtained from four different tissues of the patient. An allelic imbalance reflecting the overrepresentation of paternal alleles over those of maternal origin is profoundly observed in the resected pancreas and peripheral blood leukocytes (PBL) and to a lesser extent in buccal cells. In contrast, the DNA profile in hair follicles shows a practically normal biparental contribution.

DNA profiling was performed to determine the proportions of the androgenetic cell line in four different gDNA samples obtained from the patient. Semi-informative (one shared allele between father and mother) and informative (no shared allele between parents) genotypes were identified in the following 10 loci: THO1 (11p15.5), D3S1358 (3p25.3), VWA (12p13.31), D21S11 (21q21.1), D19S433 (19q12), D2S1338 (2q35), D16S539 (16q24-qter), CSF1PO (5q33.3-34), FGA (4q28), and D18S51 (18q21.3). Analysis of X- and Y-related amelogenin amplicons showed that there was an XX-sex chromosomal complement in all analyzed DNA samples. Additionally, only a single set of paternal and maternal alleles was identified across the four gDNA samples obtained from the patient; the proportion estimates of the androgenetic cell lineage are summarized in Table 1. Representative electropherograms illustrating three semi-informative and informative STR genotypes are shown in Figure 4. This analysis revealed that there was an excess of androgenetic cell populations (P1P1) compared to the biparental ones (M1P1), particularly in the resected pancreas (74%) and peripheral leukocytes (84%) (Table 1). These findings correlated with the WES results showing ~92% ROH. Due to the lower proportion of the androgenetic cell line in buccal cells (47%), this sample, along with gDNA samples from both parents, was selected for an additional WES trio analysis. The results revealed a GWpUPIDM proportion of ~62% (Figure 2), which resembles the 47% estimate obtained by DNA profiling. The second WES analysis additionally identified an allelic frequency of ~75% for the paternally inherited splicing variant NM_000158.4:c.555+1G>T [ClinVar: 632422; dbSNP: rs759707498] in the *GBE1* gene (3p12.2, OMIM *607839), which is associated with glycogen storage disease type IV (GSDIV, OMIM #232500). This variant had exhibited a higher allelic frequency (95%) in the first WES performed using gDNA from peripheral blood leukocytes.

## 3. Discussion

Here, we report the first Mexican/Latin American female patient diagnosed with GWpUPIDM. This individual initially presented with non-syndromic hyperinsulinism and severe neonatal hypoglycemia, requiring subtotal pancreatectomy. The diagnosis of GWpUPIDM was incidentally established through WES, which revealed ~92% of ROH in gDNA from peripheral blood leukocytes. DNA profiling using 15 autosomal STR markers confirmed that there was a single haploid paternal genome in both androgenetic and biparental cell lines, suggesting that the GWpUPIDM originated from the fertilization of a single haploid oocyte by a single haploid sperm carrying an X chromosome. This was probably followed by tripronuclear zygotic division, leading to the formation of a normal biparental cell lineage (M1P1), while the androgenetic lineage (P1P1) arose due to haploid rescue by a second endoreplication event of the male pronucleus [2]. A zygotic 69,XXX triploidy rescue could be considered a less plausible mechanism due to the key involvement of dispermic fertilization in its etiology, which generally leads to the presence of two different paternal haploid genomes among biparental and androgenetic cell lineages [2].

Previous reports indicated that GWpUPIDM presents highly variable proportions of mosaicism across different tissues (mean 5–85%), which is thought to contribute to the broad clinical heterogeneity observed in these patients [1]. However, assessment to determine the degree of biparental and isodisomic androgenetic somatic mosaicism among different tissues is quite variable among the reported patients [9], and there is no consensus to establish a minimum number of cell types to be analyzed to prove GWpUPIDM [19]. For example, as reviewed by Spier et al. [9], in 20 female patients with proven GWpUPIDM, the number of tissues analyzed ranged from only one (i.e., lymphocytes, placenta, or kidney) to five (lymphocytes, skin, buccal cells, adrenal tissue, and cecal appendix). Similarly to our patient, four or more tissue types were analyzed in only 20% of cases (N = 4/20), and hyperfunctioning pancreatic tissue was evaluated in only three of them. Despite their accessibility, buccal cells (N = 3/20 cases) and urinary sediment (N = 1/20 cases) were studied in a minority of these patients. In contrast to our patient, none of them had a genetic assessment of purely ectodermal-derived tissue, such as hair follicles.

The high proportion of the androgenetic cell line (65–85%) in the hyperplastic pancreatic tissue of our patient is consistent with findings in other patients, where this predominance has been correlated with hyperinsulinemia, likely driven by imprinting disturbances in the 11p15 region caused by paternal uniparental disomy [1,2,12,15,20]. Despite the variable degree of mosaicism between the paternal uniparental and biparental cell lineages, our 8.5-year-old patient had not developed any manifestation of another MPID or evidence of neoplasia. Skin pigmentary anomalies are not considered a distinctive feature of GWpUPIDM [1]; however, a Blaschkoid mixture of lighter and darker dermal pigmentation was reported in an affected female newborn carrying very low-grade GWpUPIDM mosaicism in her skin (<5%) [2]. We were unable to determine whether PMD, which is reported in ~86% of GWpUPIDM cases [1], was present during gestation; however, our patient shares key prenatal findings with previous reports, including a history of pre-eclampsia and preterm delivery [1,21].

DNA profiling revealed a higher proportion of GWpUPIDM cells in tissues derived mainly from the mesoderm (peripheral blood leukocytes: 75–89%) and endoderm (pancreas: 65–85%) (Table 1). In contrast, a significantly lower proportion of the androgenetic cell line was seen in the mainly ectoderm-derived tissue buccal cells (34–58%) and hair follicles (0–4%), the latter of which predominantly contained normal biparental cells. These findings allow us to speculate that the nervous central system in our patient may predominantly consist of normal biparental cells, potentially avoiding a critical threshold for the manifestation of AS features (i.e., neurodevelopmental delay, seizures, facial dysmorphias, ataxic gait, moria, etc.). Interestingly, a previously reported GWpUPIDM patient (No. 1) affected by BWS and AS stigmata showed a slightly higher proportion of the androgenetic cell line in ectoderm-derived samples (5–20%), including leg and abdominal skin [1]. However, methylation and genotypic profiling in other GWpUPIDM patients with both BWS and AS stigmata have been performed solely on mesoderm-derived tissues, such as leukocytes and arm-skin fibroblasts [11]. Given these observations, a correlation between the degree of GWpUPIDM mosaicism in ectodermal/neuroectodermal tissues and the presence or absence of AS features warrants further investigation. Additionally, it is noteworthy that our patient did not exhibit any phenotypic features associated with MPIDs beyond the 11p15 region. For instance, she lacked the thoracic abnormalities commonly observed in patUPD14, such as a small bell-shaped thorax, ‘coat-hanger’ ribs, and a narrow chest wall. Similarly, she did not present hypocalcemia, hyperphosphatemia, or bone abnormalities related to renal tubule resistance to parathyroid hormone, all of which are characteristic of patUPD20 [22]. However, it remains to be determined whether phenotypic manifestations related to patUPD6, such as diabetes mellitus caused by impaired insulin secretion in the absence of autoantibodies, may develop later in our patient’s life. Notably, 50–80% of patients with transient neonatal diabetes mellitus type 1 associated with patUPD6 eventually progress to diabetes mellitus in adolescence or adulthood [23]. This is particularly relevant considering our patient’s history of subtotal pancreatectomy, which may predispose her to developing an insulin secretion impairment later in life.

The limited number of GWpUPIDM patients reported to date and the highly variable degree of mosaicism hinders the establishment of standard guidelines for cancer surveillance in these patients [24]. It has been estimated that BWS patients with GWpUPIDM have a 50% risk of developing typical BWS-associated neoplasms and a 69% lifetime risk of developing tumors [1]. Interestingly, consistent with our patient’s neoplasm-free status at age 8.5, two previously reported GWpUPIDM patients who lacked BWS stigmata had reportedly remained neoplasm-free until early adolescence [16,25]. However, given the high overall risk for neoplasia development (69–79%) observed in GWpUPIDM [1,4], our patient will require strict outpatient pediatric oncology surveillance [16].

The etiology of a possible undiagnosed hepatopathy in our patient also remains unclear and warrants further investigation. It will be essential to rule out an underlying and unmasked autosomal recessive *GBE1*-related disorder, given the presence of the pathogenic variant NM_000158.4(*GBE1*):c.555+1G>T, which is an extremely low-frequency allele that has been almost exclusively documented in Admixed American populations (0.006254%) according to gnomAD v2.1.1 (https://gnomad.broadinstitute.org/variant/3-81698946-C-A?dataset=gnomad_r2_1, accessed on 13 March 2025). Although this variant has not been previously reported in *GBE1*-related disorders, it is predicted to disrupt RNA splicing. In our patient, this splicing variant showed a high allelic frequency in the blood (95%) and buccal cells (75%). Since significant residual GBE1 activity (20–50%) has been reported to underlie milder or even classical forms of GSDIV [26], the clinical significance of this variant in our patient remains uncertain. Up to age 8.5, our patient had not presented any hypoglycemic event beyond that addressed in the early neonatal period. Unlike other forms of glycogen storage disorders, nutritional management in GSDIV has limited effectiveness in altering disease progression, and liver transplantation remains the definitive therapeutic option for patients who progress to hepatic cirrhosis [27]. To date, our patient does not present any splenic or neuromuscular manifestations suggestive of GSDIV. Interestingly, *GBE1* splicing null variants are primarily associated with congenital forms of GSDIV but have also been linked to non-progressive hepatic forms with survival beyond 5 years of age [26]. Therefore, the long-term medical surveillance of our patient should consider the potential late-onset manifestations of GSDIV, including the possibility of adult polyglucosan body disease [28]. Genotypic and histopathological evaluation, along with measurement of glycogen branching enzyme activity in hepatic biopsy, could provide further insights to exclude or confirm a GSDIV diagnosis [27], but whether to carry it out must be considered in terms of risk versus benefit since our patient is currently asymptomatic. Indeed, confirmation in the affected tissue of a clinically relevant genotype for an autosomal recessive disorder generated by isodisomy is not always possible or justified. For example, a previously reported GWpUPIDM patient carrying a paternally inherited monoallelic *DUOX2* genotype in blood and the antecedent of congenital hypothyroidism lacked a genotypic evaluation of the thyroid tissue to correlate it with the eventual presence of an autosomal recessive trait underlying the thyroid dysfunction, as there was no indication to perform a biopsy [1].

Although GWpUPIDM remains a rare genetic condition, it is likely underdiagnosed. Its early identification through SNP-based chromosomal microarrays or WES with further confirmation through DNA profiling is crucial for ensuring strict cancer surveillance, which will ultimately improve patient survival and quality of life [1,15]. The previous reports and our present results agree that GWpUPIDM should be primarily suspected in female patients presenting with the following conditions: (a) severe congenital hyperinsulinemic hypoglycemia (presented in ~90% of cases), typically caused by pancreatic endocrine hyperplasia or nesidioblastosis [1,11]; (b) apparent isolated PMD (85.7% of cases); (c) atypical forms of BWS (8% of cases) [1], particularly those associated with other paternal imprinting disorders (20–70% of cases) [1] or a history of PMD (20% of cases) [29]; and (d) development of synchronous/metachronous malignant and benign neoplasms related to paternal uniparental disomy, which has an estimated risk of 69–79% in patients with GWpUPIDM [1,4].

## 4. Materials and Methods

### 4.1. WES and DNA Profiling to Determine GWpUPIDM

For the first WES, the salting-out method was used to obtain a gDNA sample from peripheral leukocytes collected from the patient at 8 years of age (patient ID 3bINP-012). The methodology applied, including Human Phenotype Ontology (HPO, https://hpo.jax.org/, accessed on 30 September 2025) filtering and management of incidental or secondary findings, was as previously reported [18]. The same methodology was used to perform an additional WES trio analysis using gDNA from the buccal swabs of the patient and gDNA from peripheral blood leukocytes of both parents. This second WES analysis using gDNA from patient’s buccal cells aimed to rule out the presence of additional monogenic traits.

Variant allele frequency was calculated for each SNV across the entire genome by dividing the read count of the alternate allele by the total read count, as indicated in the generated VCF file as “AD” information showing “Allelic depths for the reference and alternate alleles in the order listed”.

Additional gDNA samples from the patient were obtained from hair follicles, buccal cells, and archived formalin-fixed paraffin-embedded resected pancreatic tissue. For DNA profiling, gDNA from peripheral blood leukocytes of both parents was used alongside the patient’s gDNA samples. All gDNA extractions were performed using the salting-out method. Fifteen short-tandem repeat (STR) autosomal markers were analyzed using the Investigator IDplex Plus Kit (QIAGEN, Hilden, Germany), following the manufacturer’s standard protocol. The fragment analysis was performed with the Peak Scanner Software v. 2 (Applied Biosystems, Life Technologies Corporation, Van Allen Way, Carlsbad, CA, USA). To estimate the proportion of the androgenetic cell lineage in each of the patient’s gDNA samples, we applied the following formula: (paternal/maternal peak ratio − 1)/(paternal/maternal peak ratio + 1) × 100 = % of androgenetic cell lineage [11].

### 4.2. Ethical Considerations

The Research, Ethics, and Biosafety Institutional Committees of the National Institute of Pediatrics, Mexico, approved the protocol (Institutional number 2022/051). Before clinical imaging and molecular studies were carried out, signed informed consent documents were obtained from the patient’s parents.

## Figures and Tables

**Table 1:** Gross estimation of the proportion of androgenetic cells in four gDNA samples obtained from the patient, assessed by analyzing 10 semi-informative and informative STR markers.

Patient gDNA Sample	TH01	D3S1358	VWA	D21S11	D19S433	D2S1338	D16S539	CSF1PO	FGA	D18S51	Overall % Estimation of Androgenetic Cell Line ^1^
Peripheral blood leukocytes	88%	88%	82%	88%	81%	87%	82%	82%	89%	75%	75–89% (mean: 84%)
Pancreas	80%	80%	66%	78%	74%	NA ^2^	68%	70%	85%	65%	65–85% (mean: 74%)
Buccal cells	51%	54%	48%	58%	42%	43%	34%	46%	55%	37%	34–58% (mean: 47%)
Hair follicles	4%	0%	0%	0%	3%	0%	0%	0%	0%	0%	0–4% (mean: 0.7%)

^1^ The androgenetic cell line percentage was estimated according to the previously described formula [11]. ^2^ No PCR products were obtained from the pancreas for the high-molecular-weight STR marker, D2S1338 (380–440 pb), possibly due to the low quality or fragmentation often seen for gDNA extracted from formalin-fixed paraffin-embedded tissue.

## Data Availability

Publicly available datasets were analyzed in this study. The data can be found here: ClinVar: https://www.ncbi.nlm.nih.gov/clinvar/, accessed on 3 March 2025; dbSNP: https://www.ncbi.nlm.nih.gov/snp/, accessed on 3 March 2025; Genome Aggregation Database (gnomAD) v.2.1.1: https://gnomad.broadinstitute.org/, accessed on 3 March 2025; Online Men-delian Inheritance in Man (OMIM): https://www.omim.org/, accessed on 3 March 2025; and The National Center for Biotechnology Information (NCBI): https://www.ncbi.nlm.nih.gov/gene, accessed on 3 March 2025). The data presented in this study are available upon reasonable request from the corresponding author. The clinical and molecular data of the patient and their parents are not publicly available due to restrictions to preserve their confidentiality, which were part of the signed informed consent of each participant.
